# Computed Tomography Evaluation of Craniomandibular Articulation in Class II Division 1 Malocclusion and Class I Normal Occlusion Subjects in North Indian Population

**DOI:** 10.5402/2012/312031

**Published:** 2012-08-16

**Authors:** K. C. Prabhat, Sanjeev Kumar Verma, Sandhya Maheshwari, Ibne Ahmad, Mohd. Tariq

**Affiliations:** ^1^Department of Orthodontics and Dental Anatomy, Dr. Z. A. Dental College, Aligarh Muslim University, Aligarh 212001, India; ^2^Department of Radio Diagnosis, Aligarh Muslim University, Aligarh 212001, India

## Abstract

*Objective*. The purpose of this study is to investigate the Craniomandibular articulation morphology and position of condyle in mandibular fossae in Angle's class I normal occlusion and Angle's class II division 1 malocclusion. *Materials and Methods*. The present study was conducted on 40 subjects with 20 subjects in each group, and the computed tomography images were obtained using spiral computed tomography technique. Each measurement was compared by two-factor analysis of variance (ANOVA) while changes in anterior and posterior joint spaces were done by paired *t*-test. *Results*. Statistically significant anterior positioning of condyle (*P* > 0.05) was observed in class I normal malocclusion, and it was significant only on right side in class II division 1 malocclusion. *Conclusions*. There was no difference found in the condylar process and joint morphology between right and left sides of both Angle's Class I normal occlusion and Angle's class II division 1 malocclusion. Evaluation of the position of the condyles in their respective mandibular fossae showed concentric position with a tendency towards anterior positioning for both right and left sides of the subjects with Angle's Class I normal occlusion as well as subjects with Angle's class II division 1 malocclusion.

## 1. Introduction

The Craniomandibular articulation (CMA) is a bicondylar articulation [1], with the mouth closed; the condyle is located in a centric position in glenoid fossae. The influence of occlusion on the joint morphology is still not completely understood. Some investigators have indicated that occlusal factors are related to joint morphology [2, 3] whereas others have failed to demonstrate such a correlation [4, 5]. Actually the morphology and function are intimately related. The loads to which the CMA is subjected vary according to the subjects' dentofacial morphologies. Therefore it can be suggested that both the condyle and the mandibular fossa differ in morphology in subjects with various malocclusions [6].

In previous research, morphological characteristics of CMA particularly condyle and mandible in association with malocclusion have been studied with various imaging modalities. Diagnostic accuracy with the conventional two dimensional radiography is limited because of difficulties in imaging of the points, the location of condyle within cranial base result in bony superimposition, and structural distortion in film techniques [7]. All such difficulties of Craniomandibular articulation imaging might be eliminated by using computed tomography (CT), which allows precise visualization of anatomic details. Thus reliable data concerning morphology, irregularities, and condyle-fossa relationship can be obtained. 

Correct diagnosis of a malocclusion is essential for the planning of any orthodontic treatment. A thorough understanding of Craniomandibular articulation morphology and its spatial position in the different malocclusion and influence of orthodontic treatment on its structure during the stages of human development is still challenging job for orthodontist. If we accept the long held on belief that “function affect form” [8], the articular tissue of the Craniomandibular articulation has considerable potential for adaptation to changing functional demands; this should be kept in mind when planning orthodontic treatment [9]. The use of tomographic X-ray prior to orthodontic treatment, as well as 3-4 months prior to debonding, is helpful in evaluating the presence of irregularities within the structure of the joint and also to evaluate the patient clinical condyle position. In most cases minor changes can be made during the finishing stage [10] of the treatment to allow for the correction but no data, available in Indian population regarding skeletal morphology of the Craniomandibular joint (CMA). 

The purpose of this study is to investigatethe Craniomandibular articulation morphology and position of condyle in mandibular fossae in Angle's class I normal occlusion and Angle's class II division 1 malocclusion and to evaluate and compare the quantitative differences in joint morphology of right and left sides in north Indian population.

## 2. Materials and Methods

The present study was conducted on 40 subjects (19 male and 21 female) in the department of Orthodontics and Dental Anatomy (Dr. Z. A. Dental College, AMU, Aligarh, India). Before initiating this research work, the study was approved by board of studies of our university, and a written informed consent was obtained from each participant or their parents before inclusion in this study. Total study subjects were divided into two groups with 20 subjects in each group on the basis of inclusion criteria, detailed medical and dental history, and clinical examination. Group I included the subjects with Angle's class I normal occlusion while group II included subjects with Angle's class II division 1 malocclusion with the overjet more than 5 mm. Subjects with the age range of 14 to 25 years and those who had full set of teeth (with the exception of third molars) were included in this study. Subjects with the history of any congenital defect in dentofacial or in head and neck region, history of orthodontic/orthopaedic or surgical treatment in past, any visible facial asymmetry or subjects with dual bite tendency were not included in this study.

**Figure 1:**
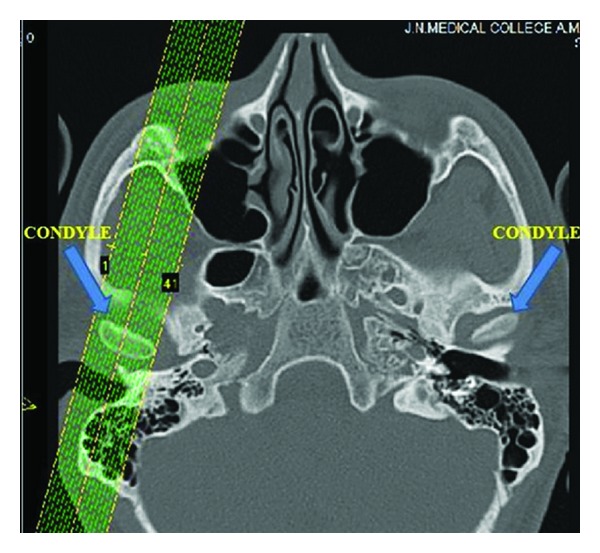
Axial pilot view of condyles (arrow) with the placement for unilateral nonorthogonal sagittal image.

**Figure 2:**
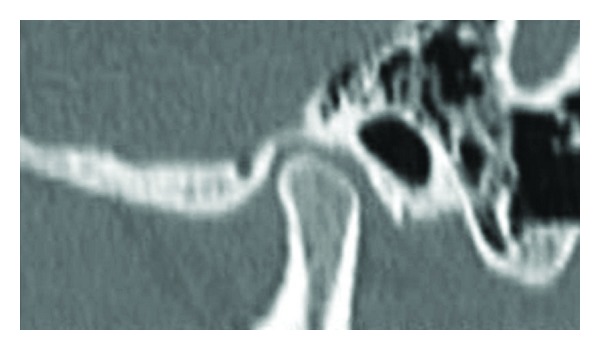
Sagittal slice computed tomography image of Craniomandibular articulation.

**Figure 3:**
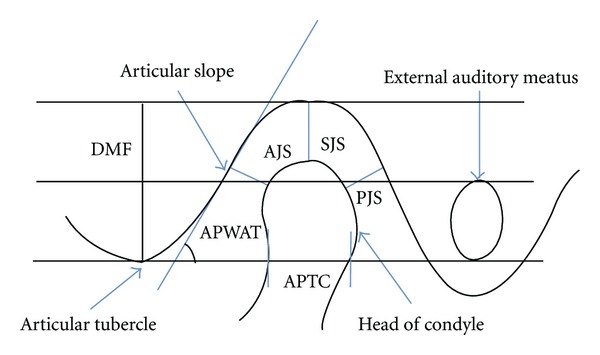
Parameters used for assessing the Craniomandibular articulation.

The computed tomography (CT) examination was done by using spiral computed tomography as described by Vitral et al. [11]. The computed tomography images were obtained with the patients in centric occlusion (maximum dental intercuspation), and their heads were positioned so that Frankfort horizontal and midsagittal plane were perpendicular to floor. The spiral CT (Somatom Balance, Siemens, Germany) was performed at 130 kV and 90 mA. We obtained 1 mm thick tomographic imaging slices spaced at 2 mm intervals, using spiral technique. Because this procedure provides images in axial plane (Figure 1), it was reformatted to produce image sagittally. The measurements were determined directly on the selected image structures (Figure 2) on the screen by two examiners for all subjects. Interexaminer reliability of the reproducibility of the measurement was assessed twice during the study in seven subjects by repeating all measurements (k-score for each measurement was never lower than 0.76). Examination of Craniomandibular articulation morphology and condyle position was done using following parameters (Figure 3) for that on both right and left sides.

(1) Depth of mandibular fossae (DMF): measured from the most superior point of the fossae to the plane formed by the most inferior point of the articular tubercle to the most inferior point of auditory meatus.
(2) Anterior joint space (AJS): expressed by shortest distance between the most anterior point of the condyle and the posterior wall of the articular tubercle.
(3) Posterior joint space (PJS): represented by shortest distance between the most posterior point of the condyle and the posterior wall of the mandibular fossae.
(4) Superior joint space (SJS): measured from the shortest distance between the most superior point of the condyle and the most superior point of the mandibular fossae.
(5) Angulation of the posterior wall of articular tubercle (APWAT): represented by the angle between the plane of posterior wall of the articular tubercle and the plane obtained from the most inferior point of the articular tubercle to the most inferior point of auditory meatus.
(6) Anteroposterior thickness of condylar head (APTC): the plane formed by the most inferior point of the articular tubercle to the most inferior point of auditory meatus divides the condyle into anterior condylar point, and posterior condylar point and the distance between these two points is the anteroposterior thickness of condyle.
(7) Percentage of anterior to posterior joint space (PAPJS) expressed as
  (1) $$\begin{array}{c} \frac{\text{anterior joint space}-\text{posterior joint space}}{\text{anterior joint space}+\text{posterior joint space}}\times 100. \end{array}$$
  (Perfect centered condyle would be expressed as 0%. A positive value indicates posterior positioning of condyle.
(8) Concentric position of condyle is expressed as anterior joint space–posterior joint space (a negative value shows anterior positioning of condyle).

Each measurement was compared by two-factor analysis of variance (ANOVA), and the significance of mean difference was done by Newman-Keuls post hoc test to evaluate the average of differences between left and right side for each element of the sample, while change in AJS and PJS was done by paired *t* test. A two-tailed (*α* = 2) probability (*P*) less than 0.05 (*P* < 0.05) was considered statistically significant. 

## 3. Results

The descriptive statistics for each measurement analyzed in the comparison of structures on left and right sides are shown in Table 1, for both the group of subjects with class I normal occlusion and subjects with class II division 1 malocclusion. The descriptive statistics for the evaluation of the concentric position of the condyles are shown in Table 2.

The mean depth of mandibular fossae was 7.93 and 7.98 mm for left and right sides, respectively, (*P* = 0.742) for class I normal occlusion and 8.79 and 8.58 mm for left and right sides, respectively, (*P* = 0.193) for class II division 1 malocclusion. The mean anterior joint spaces were 1.95 and 2.02 mm on both left and right sides, respectively, (*P* = 0.719) for class I normal occlusion and 1.87 and 1.98 mm for left and right sides, respectively, (*P* = 0.461) for class II division 1 malocclusion. The mean posterior joint spaces were 2.31 and 2.38 mm for left and right sides, respectively, (*P* = 0.438) for class I normal occlusion and 2.31 and 2.25 mm for left and right sides, respectively, (*P* = 0.484) for class II division 1 malocclusion. The mean superior joint spaces were 2.42 and 2.50 mm for left and right sides, respectively, (*P* = 0.547) for class I normal occlusion and 2.51 and 2.53 mm for left and right sides, respectively, (*P* = 0.840) for class II division 1 malocclusion. The mean angulations of the posterior wall of articular tubercle were 49.73 and 48.40 degree for left and right sides, respectively, (*P* = 0.609) for class I normal occlusion and 48.60 and 48.80 degree for left and right sides, respectively, (*P* = 0.854) for class II division 1 malocclusion. The mean anteroposterior thickness of condylar head was 8.13 and 8.42 mm for left and right sides, respectively, (*P* = 0.085) for class I normal occlusion and 7.25 and 7.39 mm for left and right sides, respectively, (*P* = 0.402) for class II division 1 malocclusion. The percentage of anterior to posterior joint spaces was −8.42 and −8.54% for left and right sides, respectively, (*P* = 0.003) for class I normal occlusion and −10.15% and −5.46% for left and right sides, respectively, (*P* = 0.290) for class II division 1 malocclusion.

## 4. Discussion

 We assessed Craniomandibular articulation (CMA) morphology by computed tomography (CT) as CT has been shown to be ideal tool for CMA assessment because it allows very accurate evaluation of skeletal anatomic details [12, 13] without superimposition of any other structure [14]. CT scanning has following advantages [15] over conventional radiography as follows It provides three-dimensional information in the form of thin slices, so internal structures can be evaluated: this eliminates the superimpositions. CT can detect density differences in the tissues of less than 1% while conventional radiography depicts the tissues that show a density differences at least 10%. The sagittal slice CT is the most appropriate for assessing the condyle-fossae relationship [15]. In the present study CMA morphology was studied in the subjects with the age ranging from 14 to 25 years as it is reported in literature [16, 17] that mandibular fossae attain their adult sizes before the age of 8 and did not show significant change after this age. 

We studied CMA morphology in subjects with Angle's class I normal occlusion and subjects with Angle's class II malocclusion because class II malocclusion has been described as most frequent treatment problem in orthodontic practice [18], and till the date of the planning of the study we did not find any CT study of Craniomandibular articulation morphology reported in literature on subjects with Angle's class I normal occlusion. Hence this finding of present study could be a valuable reference for evaluation and comparison of TMJ morphology in different malocclusions in north Indian population.

Our result, for the depth of mandibular fossae both in class I normal occlusion and class II division 1 malocclusion were not statistically significant (*P* > 0.05) when the right and left sides were compared (Table 1). Nonsignificant (*P* > 0.05) results were also obtained when the condyle-fossae relationship was assessed for right and left sides through the measurements of the anterior, superior, posterior joint spaces and anteroposterior thickness of condylar head (Table 1) in both class I normal occlusion and class II division 1 malocclusion subjects. The lack of asymmetry in these measurements is similar to those previous studies in which the same methodology was applied for the different types of the malocclusion [11, 19, 20]. Since the sagittal evaluation showed no significant differences regarding condylar dimension and positioning, the asymmetry in the posterior articular space can be explained by different dimensions of mandibular fossae. 

Our result for the angulation of the posterior wall of articular tubercle (Table 1) is similar to the study conducted by Vitral et al. [11] and they found value of 51.37° for class I and 52.40° for class II side in class II division 1 subdivision malocclusion. Our result for APWAT is not in line with the study conducted by Christiansen et al. [21]. However, their results cannot be compared with our study because they did not specify the plane from which the angle was measured. 

We assessed the concentric positioning of the condyle by comparing the differences in AJS and PJS (Table 2). We found that AJS for both right and left sides was significantly (*P* < 0.01) smaller than PJS in the subjects with class I normal occlusion. While the subjects with class II division 1 malocclusion AJS were significantly (*P* < 0.01) smaller than PJS on the left sides, no significant difference was found on right sides. Therefore findings of the present study suggest anteriorly position of condyle in mandibular fossae. Initial studies conducted for the evaluation of condylar concentricity showed centralization of condylar process [22–24]. Pullinger et al. [25] also showed that anterior positioning of condyle is the feature of class II division 1 malocclusion sample. Vitral et al. [11] with the same methodology used in our study found a more anterior condylar position bilaterally in subjects with class II division 1 subdivision malocclusion. The recent studies [19, 20] that used more sophisticated diagnostics and imaging techniques did not confirm the findings of older studies, which described the centralized condylar positioning in relation to the mandibular fossae. Result of our finding regarding the concentric positioning of condyle is similar to the previous study conducted by Cohlmia et al. [26]. They reported that left condyle was more anteriorly positioned than the right in a sample of the patients with malocclusion after comparing the anterior articular space for right and left TMJ. According to these authors this asymmetry could be related to normal cranial base asymmetries and side preference during the mastication.

 In present study on comparing the mean PAPJS (Table 1) at the left and right sides, did not differ significantly (*P* > 0.05) in both class I normal occlusion and class II division 1 malocclusion. This result showed anterior positioning of condyle in mandibular fossae. This equation (suggested by Pullinger) determined the percentage of anterior or posterior displacement of condyle, with concentricity as a reference. A score of −12% to +12% of posterior to anterior joint space is used to describe concentricity of condyle in mandibular fossae. Hence the overall findings of this study suggest that both right and left condyles were centered in mandibular fossae with a tendency towards anterior positioning. The condylar-fossae morphology, as it is presented here in our study, was assessed from the sagittal slice computed tomogram as a step towards the objective measurement of the shape of the condyle and fossae by using methods that are applied in the wider field of biologic sciences. There is no doubt that this methodology is very simple and sophisticated. Further evaluation of the relationship between shape and function of the Craniomandibular articulation is needed in various groups of malocclusions.

## 5. Conclusion

From the present study, the following conclusion can be drawn.

1. There were no differences found in the condylar process and joint morphology between right and left sides of both Angle's class I normal occlusion and Angle's class II division 1 malocclusion.
2. Evaluation of the position of the condyles in their respective mandibular fossae showed concentric position with a tendency towards anterior positioning for both right and left sides of the subjects with Angle's Class I normal occlusion as well as subjects with Angle's class II division 1 malocclusion.

## Figures and Tables

**Table 1:** Statistical analysis: structures on left and right sides for both Class I normal occlusion and Class II Division 1 malocclusion.

Parameters	Class I normal occlusion (*n* = 20)			Class II Division 1 malocclusion (*n* = 20)		
	Left	Right	*P* value	Left	Right	*P* value
DMF (mm)	7.93 ± 0.93	7.98 ± 1.06	0.742	8.79 ± 0.69	8.58 ± 0.58	0.193
AJS (mm)	1.95 ± 0.31	2.02 ± 0.33	0.719	1.87 ± 0.18	1.98 ± 0.32	0.461
PJS (mm)	2.31 ± 0.39	2.38 ± 0.29	0.438	2.31 ± 0.37	2.25 ± 0.55	0.484
SJS (mm)	2.42 ± 0.37	2.50 ± 0.52	0.547	2.51 ± 0.63	2.53 ± 0.68	0.840
APWAT (degree)	49.73 ± 5.31	48.40 ± 3.48	0.609	48.60 ± 2.26	48.80 ± 2.83	0.854
APTC (mm)	8.13 ± 1.61	8.42 ± 1.35	0.085	7.25 ± 1.01	7.39 ± 1.12	0.402
PAPJS (%)	−8.42 ± 11.21	−8.54 ± 7.29	0.993	−10.15 ± 7.62	−5.46 ± 14.31	0.446

**Table 2:** Statistical analysis: concentric position of condyle in Class I normal occlusion and Class II Division 1 malocclusion.

Groups	Sides	Anterior joint space (AJS)	Posterior joint space (PJS)	Anterior joint space–Posterior joint space (AJS-PJS)	Paired Student's *t*-test	*P* value
Class I normal occlusion	Left	1.95 ± 0.31	2.31 ± 0.39	−0.37 ± 0.46	3.09	0.0008
	Right	2.02 ± 0.33	2.38 ± 0.29	−0.36 ± 0.31	4.55	0.0005
Class II Division 1 malocclusion	Left	1.87 ± 0.18	2.31 ± 0.37	−0.44 ± 0.37	4.58	0.0004
	Right	1.98 ± 0.32	2.25 ± 0.55	−0.27 ± 0.71	1.46	0.1655
